# Lymphoma and Myeloma Cell Resistance to Cytotoxic Agents and Ionizing Radiations Is Not Affected by Exposure to Anti–IL-6 Antibody

**DOI:** 10.1371/journal.pone.0008026

**Published:** 2009-11-30

**Authors:** Angélique Gougelet, Adeline Mansuy, Jean-Yves Blay, Laurent Alberti, Claudine Vermot-Desroches

**Affiliations:** 1 Unité INSERM U590 équipe Cytokines et Cancer, Centre Léon Bérard, Lyon, France; 2 Conticanet Consortium (FP6-06188),IID-Biotech, Dardilly, France; 3 EORTC, Brussels, Belgium; 4 IDD-Tech, Dardilly, France; University of Minnesota, United States of America

## Abstract

**Background:**

Production of high levels of IL-6 is often correlated with resistance to cytotoxics or ionizing radiations, in cancer cell lines as in various cancer patients. We investigated whether monoclonal antibodies directed against IL-6 may enable to reverse resistance of cancer cell lines.

**Methodology/Principal Findings:**

We exposed ten haematological cancer cells from lymphoma, myeloma, or leukemia origins to cytotoxics or ionizing radiations and assessed the effects of anti–IL-6 antibody addition on cell proliferation, apoptosis, or IL-6 signaling. A strong correlation between IL-6 secretion, measured by ELISA, and resistance to doxorubicin as ionizing radiations was observed in the multiple myeloma U266 and the Burkitt's lymphoma Daudi and Namalwa cells. Although an anti–IL-6 antibody combined to both treatments efficiently blocked IL-6 signaling in U266 cells, expressing the IL-6 receptor gp80, it did not increase treatment-induced anti-proliferative and pro-apoptotic effects on these cells, as well as on Daudi and Namalwa cells. This lack of effect could be related to diverse factors: 1) a higher release of the soluble form of IL-6 receptor gp80 in response to doxorubicin and irradiation from all cell lines, 2) an impaired level of the IL-6 pathway inhibitor SOCS3 in Daudi cells, and 3) an increased release of IL-10 and TNFα, two cytokines involved in cell radio- and chemoresistance.

**Conclusions/Significance:**

These data support the fact that IL-6 is not the preponderant actor of cell resistance to cytotoxics and ionizing radiations, which seems to be regulated by a complex network of proteins.

## Introduction

Interleukin-6 (IL-6) is a key cytokine mainly produced by a broad variety of cell types including monocytes, fibroblasts, endothelial cells, and epithelial as haematological tumour cell lines [1]. IL-6 is particularly involved in immune response, inflammation and in haematopoiesis by controlling proliferation and maturation of B and T cells and differentiation of cytotoxic T cells, macrophages and megakaryocytes. Besides, IL-6 promotes the proliferation of haematological malignancies (leukemia, lymphoma and myeloma), and solid tumours (breast and renal adenocarcinoma or Kaposi sarcoma) [1], [2], through an intracrine, autocrine and paracrine mode of action [3], [4]. Finally, a high IL-6 serum level is often associated to worse progression-free survival and overall survival in Non Hodgkin Lymphoma [5], myeloma [6], renal carcinoma and breast adenocarcinoma [7], [8].

IL-6 exerts its biological effects through binding to its ligand-binding receptor gp80 and thereupon to two signal transducing receptor subunits gp130 [9]. IL-6 binding results in gp130 dimerization and in the subsequent activation of Janus kinases, which activate in turn gp130 through phosphorylation of its intracellular domain. Phosphorylated tyrosines in gp130 form docking sites for SH2 domain containing proteins like activators of transcription STAT1 and STAT3 [10]. Optimal activation of STAT3 requires phosphorylation of its tyrosine 705 along with phosphorylation of the serine 727 residue. In parallel, gp130 activation results in the activation of Raf/Ras/MEK cascade, and finally in phosphorylation and subsequent activation of p42 and p44 [11]. Finally, tyrosine motifs in gp130 are also crucial for recruitment of the feedback inhibitor SOCS3 (suppressor of cytokine signaling 3), which modulate IL-6 signaling *via* Jak inactivation and inhibition of STAT3/gp130 contact [12].

Despite the improvement of current therapies, most cancers in advanced phase develop intrinsic or acquired resistance [13]. *In vivo* in man, resistance to cytotoxics is associated with the overproduction of several cytokines, in particular IL-6, IL-10, VEGF (Vascular Endothelial Growth Factor) and TNF-α (Tumour Necrosis Factor) [7], [14]–[17]. *In vitro*, IL-6 protects M1 myeloid leukemia cells against apoptosis induced by cytotoxic drugs [18], but also renal carcinoma cells (RCC), erythro-leukemic cells and myeloid leukemia cells against the effects of cisplatin [16], [19], [20]. IL-6 may also be responsible for tumour cell resistance to ionizing radiations in B cells or oral cancer cells [21], [22], through a STAT3-dependent pathway [21]. Interestingly, in addition to the presence of baseline levels of IL-6, an overproduction of this cytokine in serum often occurred following irradiation of patients with breast, sarcoma, liver or head and neck cancers [23], [24]. Monoclonal antibodies blocking molecules which protect cells against cytotoxic agents, may enable reverting resistance [25]. Anti-IL-6 antibodies emerge as a new therapeutic adjuvant option for patients attained of haematological cancers, as strengthened by preclinical works using anti-IL-6 antibodies with cis-diamminedichloroplatinium (CDDP) in RCC [16] or melphalan in advanced multiple myeloma [26].

In the present work, we investigated the capacity of a targeted therapy directed against IL-6 to modulate cellular resistance to radiotherapy and chemotherapy. Following the precise identification of tumour cell dependence for IL-6 (expression of IL-6 and its receptor IL-6R) in a panel of ten cell lines (of myeloma, leukemia or lymphoma origins), we determined their individual response to different doses of radiations and chemotherapeutic drugs. Thus, we selected a panel of four cells with differential chemo- and radiosensitivity and various expression levels of IL-6 as IL-6R, and tested the effect of anti-IL-6 antibodies on cell proliferation, apoptosis and IL-6-dependent cell signaling in combination with cytotoxics.

## Results

### Interleukin-6, IL-6 receptor expression, and resistance to cytotoxics

The mRNA and protein expression of IL-6 and its two receptor subunits gp130 and gp80 was assessed in a panel of nine haematologic tumour cell lines, using flow cytometry, ELISA and RT-PCR, comparing with the triple positive control U266 cells. All cell lines expressed both soluble and membrane forms of gp130, except U937 and BJAB cells which expressed membrane gp130 only (Table 1). U937, BJAB and RPMI8226 cells expressed both soluble and membrane bound gp80 forms. Daudi and BL-36 cells also expressed the soluble form of gp80, while Raji and Namalwa were negative for both gp80 forms. Only the four Burkitt's lymphoma Raji, Daudi, BL-36 and Namalwa cells expressed IL-6; this latter expressed very low level IL-6, weakly detectable by PCR (Figure S1).

**Table 1:** Detection of membranous and soluble forms of gp130 and gp80 and detection of IL-6 in tumour cell lines.

	Membranous form (% positive cells)			Soluble form (pg/mL)		
	gp130	gp80	IL-6	gp130	gp80	IL-6
U266	71	91	64	322±101	6739±2372	34±23
RPMI8226	26	24	0	1005±475	4706±1410	0
U937	75	80	0	0	1878±567	0
Rs4; 11	72	1	0	62±78	0	0
Raji	82	66	57	94±55	0	13±12
BL-36	87	1	51	422±288	358±148	8±7
Daudi	74	3	68	66±97	169±154	13±7
Ramos	89	3	0	371±200	0	0
BJAB	70	92	0	0	665±258	0
Namalwa	78	3	59	66±92	0	8±5

Membrane staining of gp130 and gp80 and intracellular staining of IL-6 were performed on 2×10^5^ cells as described in materials and methods with 1 µg/mL primary antibody. Results were expressed as the mean % of positive cells of two independent experiments realized in duplicate. IL-6, soluble gp130 (sgp130) and sgp80 were measured by ELISA on 100 µL supernatants as described in materials and methods. Results were expressed as the mean±SD of three independent experiments realized in duplicate (pg/mL).

**Figure 1:**
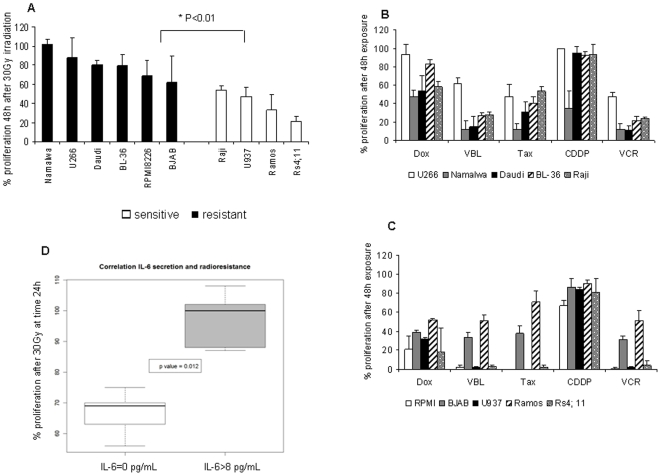
Cell response to anticancer treaments. (A) Response to radiation exposure. Following an irradiation at 30Gy, cells were plated in 96 well plates at 5,000 cells/well. An ATP-based proliferation assay was conducted 48 h after, as described in materials and methods. Results were expressed as the percentage of proliferation = number of irradiated cells at 48 h/number of cells at t0 in control conditions ×100±S.D, and represented the mean of three experiments realized in duplicate. The p value was determined according to a paired T-test * p<0.05. (B,C) Response to cytotoxics. Cells were exposed to doxorubicin, vinblastine and taxol at 0.1 µg/mL, to vincristine and cisplatin at 1 µg/mL in a 96 well plate at 5,000 cells/well for 48 h. An ATP-based proliferation assay was conducted as described in materials and methods. Results were expressed as the % of proliferation = number of treated cells at 48 h/number of cells at t0 in control conditions ×100±S.D and represented the mean of two independent experiments realized in duplicate. (D) Correlation between IL-6 secretion and radioresistance. This box-plot compiled data from ELISA assay described in Table 1 and % proliferation of cells 24 h after an irradiation at 30Gy. We divided cells into two groups, cells which did not secrete IL-6 (IL-6 = 0) and cells producing IL-6 (IL-6>8, minimal value obtained for BL-36 and Namalwa cells). The p value was calculated according a Wilcoxon statistical test.

Cell proliferation was measured 24, 48 or 72 h following exposure to ionizing radiations (7, 15 and 30Gy, as previously reported [27], [28]), using an ATP-based proliferation assay and a thymidine incorporation assay. Results with both tests were consistent as shown in Figure S2 for Daudi cells. For convenience, we used ATP based assay in the following experiments. Raji, Ramos, U937 and Rs4; 11 cells were classified as sensitive to radiation exposure since proliferation was reduced by 50% at least 48 h following an exposure to 30Gy (Figure 1A). BJAB, RPMI8226, U266, BL-36, Namalwa and Daudi cells were resistant to any dose of radiations since more than 50% of cells continue to proliferate 48 h after irradiation. Cells were also exposed to doxorubicin, vinblastine and taxol at 0.01, 0.1 and 1 µg/mL or to vincristine and cisplatin at 0.1, 1 and 10 µg/mL for 24, 48 or 72 h, as previously described [17], [29], [30]. All cell lines were resistant to a 48 h exposure to cisplatin at 1 µg/mL, except Namalwa cells (Figure 1B). Most of cell lines were highly sensitive to vinblastine, vincristine and taxol, when U266, Ramos and BJAB cells were more resistant to these drugs (40–50% proliferation after 48 h exposure). U937, Rs4; 11, BJAB and RPMI8226 cells were highly sensitive to doxorubicin (Figure 1C and Table 2), comparing with Raji, Ramos, Namalwa and Daudi cells which displayed a partially resistant pattern. U266 and BL-36 cells were strongly refractory to this drug (Figure 1B).

**Table 2:** Synthesis of cell sensitivity to cytotoxics.

	U266	Namalwa	Daudi	BL-36	Raji	RPMI 8226	BJAB	U937	Ramos	Rs4; 11
Dox	−	+	+/−	−	+/−	+	+	+	+/−	−
CDDP	−	+	−	−	−	−	−	−	−	−
VBL/Tax/VCR	+/−	+	+	+	+	+	+/−	+	+/−	+

Cells were exposed to doxorubicin, vinblastine and taxol at 0.1 µg/mL, to vincristine and cisplatin at 1 µg/mL in a 96 well plate at 5,000 cells/well for 48 h. An ATP-based proliferation assay was conducted as described in materials and methods. (−): resistance = proliferation >50%, (+/−) intermediate sensitivity = 30%< proliferation <50%, (−) sensitivity = proliferation <30%.

Thus, cells producing IL-6, e.g. U266, BL-36, Namalwa and Daudi cells, were significantly more resistant to the anti-proliferative effect of cytotoxics, as shown in Figure 1D (p≪0.05). To further investigate this question, four cell lines were selected for subsequent experiments: 1) the multiple myeloma U266 cells as a reference, expressing IL-6 and both IL-6R chains, and moderately affected by ionizing radiations as chemotherapy, 2) Daudi, a cell line resistant to radiotherapy, poorly affected by doxorubicin and cisplatin, expressing IL-6, gp130 and soluble gp80, 3) Namalwa cell line, similar to Daudi cells, except for its higher sensitivity to cisplatin and its lack of gp80, and finally 4) U937 cells, sensitive to cytotoxics and radiotherapy, which did not secrete IL-6 but expressed both membrane IL-6 receptor chains (Figure S1).

### IL-6 signaling inhibition by anti–IL-6 antibody in U266 cells

**Figure 2:**
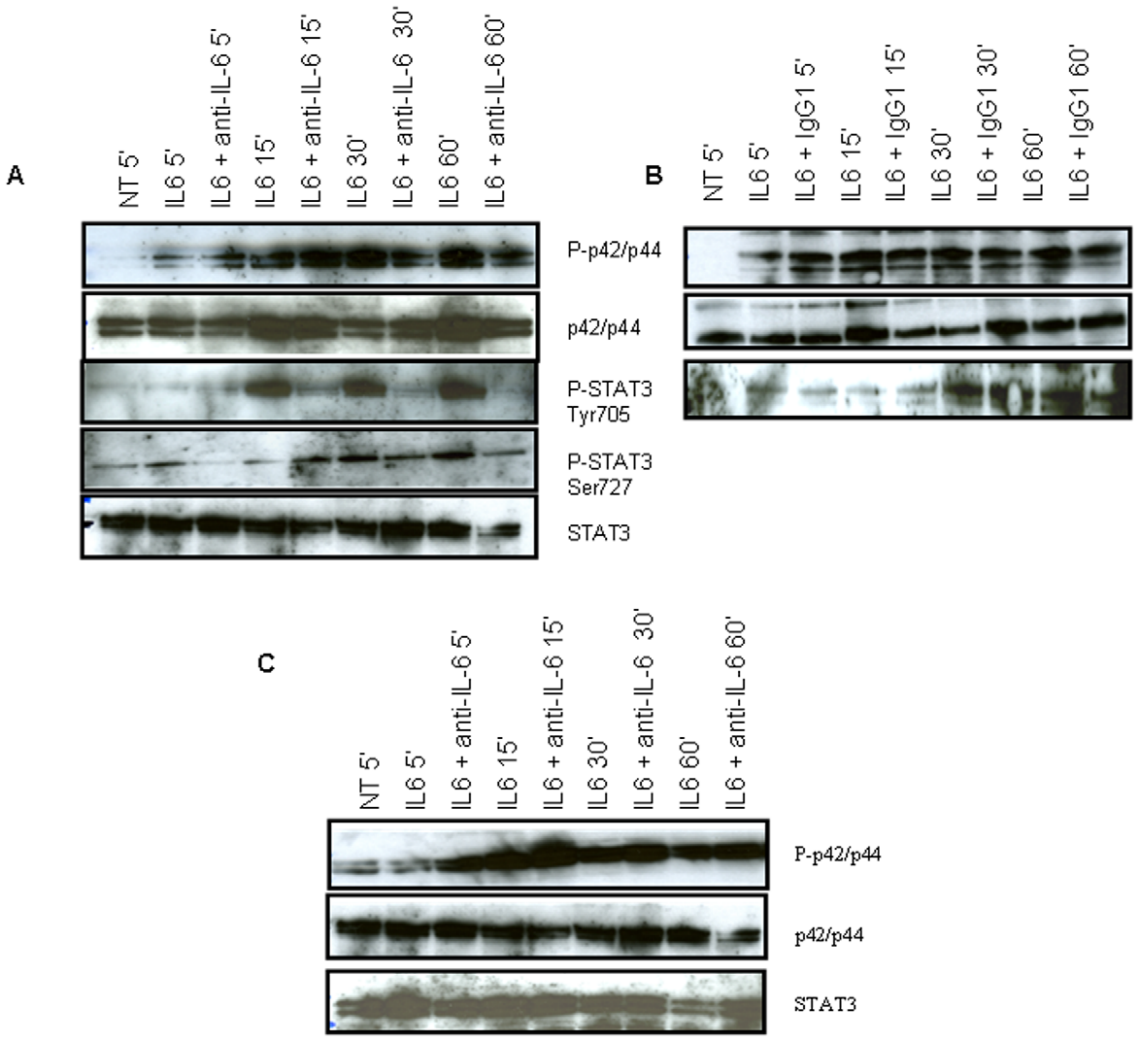
IL-6–induced phosphorylation of STAT3 and p42/p44 was efficiently reduced by an anti–IL-6 in U266 but not in Daudi cells. (A,B) U266 cells were exposed to 10 ng/mL IL-6 or not (NT) with or without 10 µg/mL anti-IL-6 (A) or IgG1 control (B) for 5, 15, 30, or 60 min. 30 µg of total proteins were analyzed with anti-phospho-STAT3 (Ser727 or Tyr705), phospho-p42/p44, STAT3 or p42/p44 at a dilution 1/1,000 and revealed with anti-rabbit-HRP 1/100,000, as described in materials and methods. (B) Daudi cells were exposed to 10 ng/mL IL-6 or not (NT) with or without 10 µg/mL anti-IL-6 for 5, 15, 30, or 60 min. 50 µg of total proteins were analyzed with phospho-p42/p44, STAT3, or p42/p44 at a dilution 1/200 and revealed with anti-rabbit-HRP 1/50,000, as described in Materials and Methods.

Following these observations, we assessed the effect of a brief exposure of U266 cells (5, 15, 30 or 60 min) to an antibody raised against IL-6, in the presence of exogenous IL-6, on the main actors of IL-6 pathways, STAT and p42/p44. As shown in Figure 2A, exposure to IL-6 resulted in p42/p44 phosphorylation after 5 min. A maximal STAT3 phosphorylation was observed on Tyr705 (p-STAT3-Tyr705) at 15 min, while Ser727 phosphorylation peaked at 30 min. The level of p42/p44 and STAT3 was marginally affected by IL-6. The addition of anti-IL-6 antibodies successfully blocked STAT3 and p42/p44 phosphorylation, particularly after 30 and 60 min exposure. The isotype-matched control antibody had no impact on the phosphorylated form of p42/p44 and STAT (Figure 2B).

**Figure 3:**
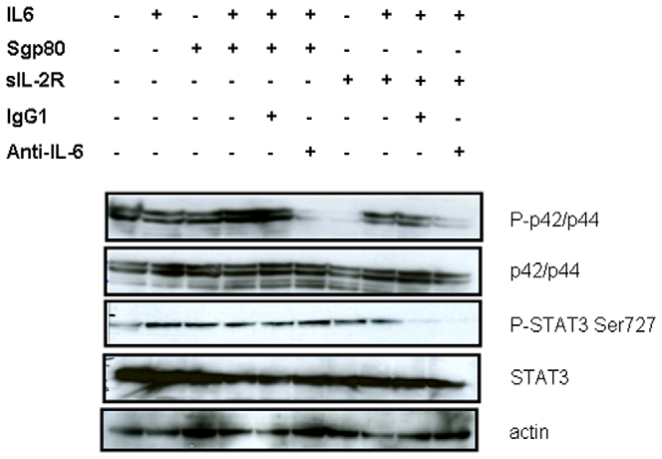
IL-6 induced p42/p44 phosphorylation was efficiently reduced by an anti–IL-6 antibody in the presence of exogenous gp80 in Daudi cells. Daudi cells were exposed to 10 ng/mL IL-6 +/− 50 ng/mL soluble gp80 or IL-2R with or without 10 µg/mL anti–IL-6 for 60 min. 50 µg proteins were analyzed with anti-phospho-STAT3 (Ser727 or Tyr705), phospho-p42/p44, STAT3, or p42/p44 at a dilution 1/200 and revealed with anti-rabbit-HRP 1/50,000, as described in Materials and Methods.

In contrast, the same experiments conducted on Namalwa as well as Daudi cells showed no effect of this antibody on IL-6 signaling in these cells (Figure 2C). In particular, p-STAT3-Tyr 705 was undetectable in Daudi and Namalwa cells. Thus, we hypothesized that Daudi cells were poorly responsive to IL-6 inhibition in absence of exogenous IL-6 plus gp80. We tested the concomitant exposure of Daudi cells to gp80 and IL-6 for 60 min, which was the best time observed for antibody-induced inhibition in U266 cells in Figure 2A. This revealed a weak inhibitory effect of anti-IL-6 antibody on the level of p42/p44 phosphorylated form (Figure 3). As a control, we similarly exposed Daudi cells to the same quantity of soluble form of IL-2 receptor. This induced a decrease in the phosphorylated form of p42/p44 and p-STAT3 on Ser727. As previously shown by others, IL-2 activates the MAP kinase pathway [31], as well as the phosphorylation of STAT3 [32]. By trapping IL-2, it is therefore consistent that the soluble form of IL-2R decreases IL-2R activation and downstream phosphorylation of its two STAT and p42/p44 target proteins.

### p-STAT3-Tyr705 impairment in U266 and U937 cells exposed to anti–IL-6/chemotherapy or anti–IL-6/radiotherapy combinations

**Figure 4:**
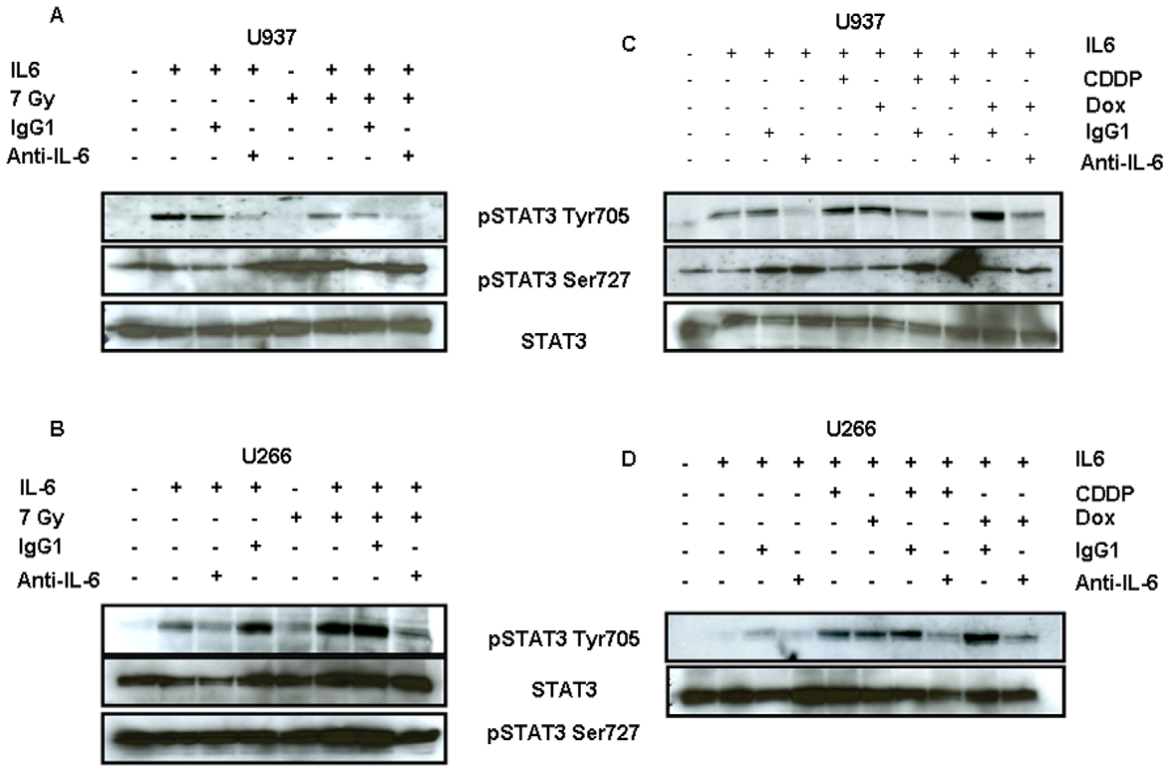
Anti–IL-6 antibody efficiently blocked IL-6 signaling in association with radiotherapy and chemotherapy on U937 and U266 cells. Following an irradiation at 7Gy, U937 (A) and U266 (B) cells were exposed to 10 ng/mL IL-6 with or without 10 µg/mL anti–IL-6 antibody for 1 h. 50 µg of total proteins were analyzed with anti-phospho-STAT3 (Ser727 or Tyr 705) or anti-STAT3 at a dilution 1/1,000 and revealed with anti-rabbit-HRP 1/50,000 as described in materials and methods. U937 (C) and U266 (D) cells were exposed to 10 ng/mL IL-6 with or without 10 µg/mL anti-IL-6 antibody in the presence of 1 µg/mL cisplatin or 0.1 µg/mL doxorubicin. 50 µg of total proteins were analyzed as previously.

We then studied the influence of chemotherapy and radiotherapy in combination with an anti-IL-6 antibody on the phosphorylated form of STAT3 on Tyr705 in U266 and U937 cells. No phosphorylation of STAT3 in control conditions and after 7Gy radiations was observed for U937 (Figure 4A) nor for U266 cells (Figure 4B). In the presence of exogenous IL-6, the anti-IL-6 antibody, or the combination with ionizing radiations efficiently blocked STAT3 phosphorylation on Tyr705 in both U937 and U266 cells. The phosphorylated form of STAT3 on Ser727 was weakly increased by radiations. Similarly, no phosphorylation of STAT3 on Tyr705 was observed after an exposure of U937 and U266 cells to 1 µg/mL cisplatin and 0.1 µg/mL doxorubicin (Figure 4B and 4D, respectively). However, STAT3 phosphorylation on Tyr705 was successfully impaired in these cells by the anti-IL-6 antibody, alone or in association with chemotherapeutic drugs, in the presence of exogenous IL-6.

### IL-6 and SOCS3 transcription in Daudi and U266 cells exposed to exogenous IL-6

**Figure 5:**
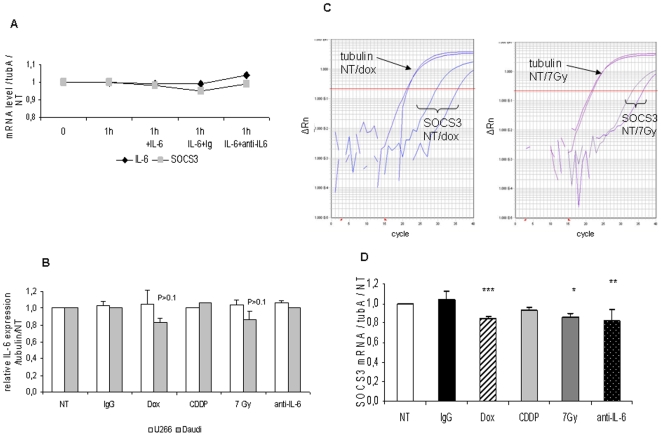
SOCS3 mRNA level is decreased in Daudi cells after 48 h exposure to doxorubicin, IL-6 antibody, and ionizing radiations. (A) Daudi cells were exposed or not to 10 ng/mL IL-6 with or without 10 µg/mL IgG1 or anti–IL-6 for 1 h. 1 µg RNA extracted from 2×10^6^ cells were subjected to RT–qPCR to measure IL-6 (⧫) and SOCS3 (▪) mRNA level as described in materials and methods. Results were presented as relative expression of gene of interest related to tubulinA expression and normalized to untreated cells at t time. (B) 1 µg RNA was subjected to RT–qPCR for IL-6 mRNA measurement following U266 (□) or Daudi cell (▪) exposure to 0.1 µg/mL doxorubicin, 1 µg/mL cisplatin, 10 µg/mL IgG1, or anti–IL-6 and 7Gy ionizing radiations for 48 h, as described in Materials and Methods. Results were expressed as IL-6 mRNA level related to tubulinA mRNA as compared to untreated cells and represented the mean of two independent experiments. The p value was determined according to a paired T-test. (C,D) 2×10^6^ Daudi cells were exposed to 0.1 µg/mL doxorubicin, 1 µg/mL cisplatin, 10 µg/mL IgG1, or anti–IL-6 and 7Gy ionizing radiations for 48 h. 1 µg RNA extracted from these cells was submitted to RT–qPCR to measure SOCS3 mRNA level. (C) raw data plot, (D) relative mRNA level. Results were presented as relative expression of SOCS3 related to tubulin A and to non-treated cells at t time and represented the mean±S.D of four independent experiments. The p value was determined according to a paired T-test * p<0.05, **<0.01, ***<0.001.

Since Daudi response to IL-6 and to anti-IL-6 antibody differed from U266 cells, we could hypothesize that IL-6 pathway is not similarly regulated in these two cell lines. It was previously shown that SOCS expression and particularly SOCS3 was regulated by a number of cytokines including IL-6 [33], and that SOCS3 modulated STAT activity [12]. After 1 h, IL-6 and SOCS3 mRNA level was similar in U266 (data not shown) and in Daudi cells (Figure 5A), in the presence of exogenous IL-6 or not, ruling out SOCS3 as responsible for the different IL-6 response in U266 and Daudi cells in these conditions.

### IL-6 inhibition has a minor effect on cell proliferation and apoptosis

**Figure 6:**
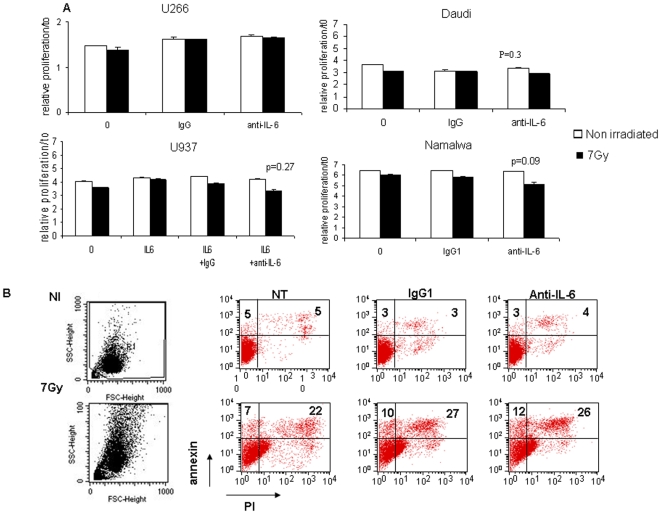
IL-6 inhibition in combination with 7Gy radiations did not inhibit U266, U937, Daudi, and Namalwa cell proliferation. (A) Cells were irradiated at 7Gy (▪) or not (□). After irradiation, cells were resuspended into fresh medium, plated in 96 well plates at 5000 cells/well and exposed to 10 µg/mL IgG1 or anti–IL-6. U937 cells were also exposed to 10 ng/mL IL-6. Cell growth was measured 48 h later for U266 and U937 cells and 72 h later for Namalwa and Daudi cells with 20 µL reagent for 10 min. Results were expressed as relative proliferation = number of irradiated cells at t time/number of cells at t0 in control conditions±S.D and represented a significant experiment among two realized in duplicate. The p value was determined according to a paired T-test. (B) Daudi radio-induced apoptosis was not improved through IL-6 inhibition. Cells were irradiated at 7Gy (lower panel) or not (NI, upper panel). After irradiation, cells were resuspended into fresh medium, plated in 6 well plates at 200,000 cells/well and exposed to 10 µg/mL IgG1 or anti–IL-6 antibody. Annexin V/PI labeling was realized on 2×10^5^ cells as described in Materials and Methods. Numbers indicated the % of cells present in each quadrant.

The four cell lines were exposed to 1 µg/mL cisplatin or 0.1 µg/mL doxorubicin in combination with anti-IL-6 antibodies for 48 h. In parallel, U266 and U937 cell proliferation was determined 48 h after exposure to a 7Gy dose of radiotherapy instead of 72 h for the more radio-resistant Daudi and Namalwa cells. In these tests, 10, 1 or 0.1 µg/mL antibodies were used with different modalities: 72 h pre-incubation, a concomitant treatment or both. Since U937 cells did not produce endogenous IL-6 (see Table 1 and Figure S1), 10 ng/mL IL-6 was added in U937 cell culture medium. The addition of anti-IL-6 antibody did not affect the proliferation of cell lines exposed to radiation therapy or chemotherapy, in any of the four cell lines, regardless of the dose and the sequence of administration. As shown in Figure 6A, an anti-IL-6 antibody exerted a minor effect on Daudi, U937 and Namalwa cell growth in association with 7Gy radiations (−20% non significant, paired T-test). Similar results were obtained for cisplatin and doxorubicin (data not shown). A lack of effect of IL-6 inhibition was also observed for BL-36 and Raji cells, the two other cell lines secreting IL-6 and resistant to radiations. A tritiated thymidine assay conducted on Daudi cells showed that an anti-IL-6 treatment significantly, although weakly, improve the cytostatic effects induced by radiations (Figure S3A) but not those induced by doxorubicin (Figure S3B). The length of treatment was not the limiting factor since an exposure of Daudi cells to anti-IL-6 for 7 days was also inefficient (Figure S3C). As a control, IL-6 involvement in cell resistance was only observed in two cases in our study: 1) IL-6 protected U937 cells from radiations; this protection was non-significantly decreased by an anti-IL-6 (Figure S4A); 2) anti-IL-6 antibody was able to increase dexamethasone-induced cytotoxic effects on Daudi cells, alone or in association with doxorubicin (Figure S4B). Finally, anti-IL-6 antibodies marginally increased the percentage of (Annexin V+) and (Annexin V/PI+) Daudi cells (Figure 6B). Similar results were obtained for chemotherapy. This was confirmed by the lack of modification of caspase activity (Figure S5). Altogether, these data suggest that IL-6 blocking in association with doxorubicin and radiations did not affect significantly the cytotoxic effects of these treatments on the cells we studied.

### SOCS3 transcriptional repression in Daudi cells following long-term exposure to radiations, doxorubicin, and anti–IL-6 antibody

We determined the level of IL-6 and SOCS3 mRNA in U266 and Daudi cells after long-term anticancer treatments by RT-qPCR. IL-6 mRNA level was stable in U266 as well as in Daudi cells after 48 h regardless of the treatment (Figure 5B). SOCS3 was also unaffected in U266 cells (data not shown). Conversely, a 48 h exposure of Daudi cells to doxorubicin, radiations or anti-IL-6 antibodies significantly decreased SOCS3 mRNA level; this was not observed with cisplatin (Figure 5C and 5D). The addition of anti-IL-6 to doxorubicin or to radiations did not further decrease SOCS3 mRNA level as compared to anticancer treatments alone (data not shown).

### IL-6 secretion in response to doxorubicin and ionizing radiations

**Figure 7:**
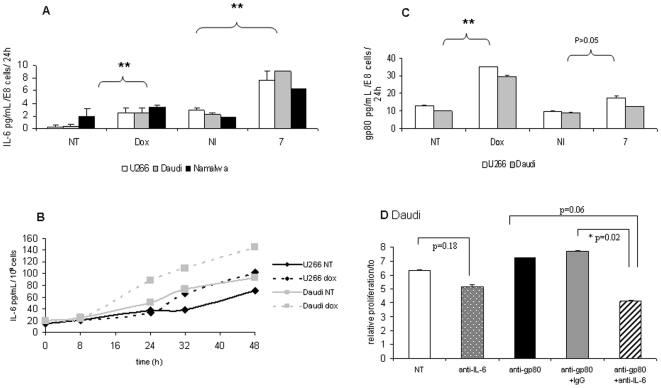
Anticancer treatments induced IL-6 secretion without affecting its mRNA level. (A) IL-6 level in cell medium was determined in 100 µL supernatants of U266 (□), Daudi (▪), or Namalwa (▪) cells, collected 72 h following an irradiation at 7Gy or not (NI) or after 48 h exposure to 0.1 µg/mL doxorubicin or not (NT), as described in materials and methods. Results were presented as (IL-6) in pg/mL secreted by 10^8^ cells at day of the assay per 24 h. (B) IL-6 secretion following exposure of U266 (⧫) or Daudi cells (▪) to 0.1 µg/mL doxorubicin for 8, 24, 32, and 48 h. Results were presented as (IL-6) in pg/mL secreted by 10^8^ cells at day of the assay per 24 h and represented the most significant experiment. The p value was determined according to a paired T-test * p<0.05, **<0.01. (C) 7Gy radiations and doxorubicin induced soluble gp80 release. Cell supernatants were collected 48 h after exposure to 0.1 µg/mL doxorubicin or not (NT) and 72 h after a 7Gy irradiation or not (NI). Sgp80 was measured by ELISA on 100 µL supernatants. Results were expressed as the concentration of gp80 in pg/mL for 10^8^ cells at day of the assay per 24 h and represented the mean of two experiments performed in duplicate. The p value was determined according to a paired T-test * p<0.05, **<0.01. (D) Daudi cells were irradiated at 7Gy. After irradiation, cells were re-suspended into fresh medium, plated in 96 well plates at 5,000 cells/well and exposed to 10 µg/mL anti-IL6 or IgG1 in the presence of 10 µg/mL anti-gp80 B-R6 or not (NT). Cell growth was measured 72 h later with 20 µL reagent for 10 min. Results were expressed as relative proliferation = number of irradiated cells at t time/number of cells at t0 in control conditions±S.D and represented a significant experiment among two realized in duplicate. The p value was determined according to a paired T-test * p<0.05, **<0.01.

An overproduction of IL-6 following irradiation of patients attained of various cancers had been previously suggested [23], [24]. In the same way, cisplatin increased IL-6 production in erythro-leukemic [19] or oral cancer cells [22]. We therefore investigated IL-6 secretion by the four selected cell lines following exposure to cytotoxics and radiotherapy. Exposure to ionizing radiations induced IL-6 release from Daudi and U266 cells: a 3-fold increase was observed for U266 and Namalwa cells and a 4-fold increase for Daudi cells (Figure 7A). Doxorubicin at 0.1 µg/mL increased by 4-fold IL-6 release from Daudi and U266 cells (instead of 1.5-fold for Namalwa cells). IL-6 measurements following kinetic experiments revealed that the induction of IL-6 secretion by doxorubicin occurred from 32 h for U266 and 24 h for Daudi cells (Figure 7B). In contrast, cisplatin had little effect on IL-6 release in all cell lines (data not shown). As previously shown in Figure 5A and 5B, kinetic experiments performed by RT-qPCR revealed no change of IL-6 mRNA level in Daudi and U266 cells during cell exposure to doxorubicin, as well as irradiation, still 48 h. Thus, doxorubicin and radiation-based treatments maintained IL-6 mRNA level through 1) a balance between mRNA synthesis and a lack of protein degradation, or 2) through mRNA stabilization before its secretion.

### Soluble gp80 release following exposure to doxorubicin and radiotherapy

In parallel to IL-6 measurements, we assessed the level of the soluble and agonist form of gp80 by ELISA in the same conditions. As for IL-6, gp80 release was found to increase in response to doxorubicin as well as ionizing radiations in U266 and Daudi cells (3-fold for doxorubicin and 2-fold for 7Gy, Figure 7C). To investigate whether the concomitant release of sgp80 could counteract the inhibitory effect of the anti-IL-6 antibody, we tested the addition of an anti-gp80 antibody to irradiated Daudi cells: this was found to weakly increase the anti-proliferative effects of radiations associated to anti-IL-6 antibodies (Figure 7D). Similar results were obtained with cisplatin, although this drug did not induce IL-6 and gp80 release from Daudi cells.

### IL-10 as TNFα secretion by U266 and Daudi cells in response to doxorubicin and ionizing radiations

**Figure 8:**
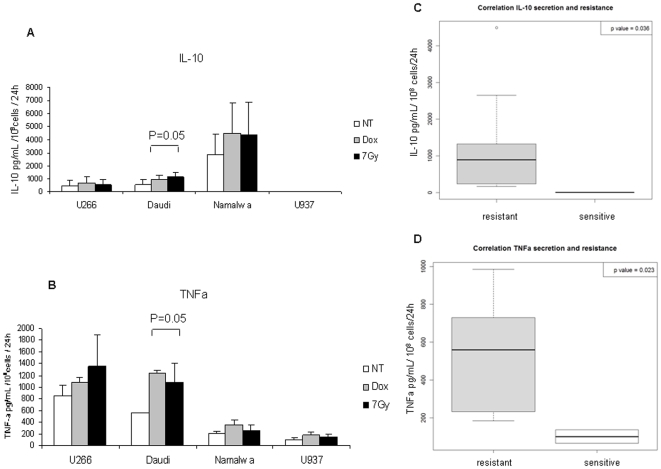
7Gy radiations and doxorubicin induced IL-10 and TNFα release from Daudi and Namalwa cells. Cell supernatants were collected 48 h after exposure to 0.1 µg/mL doxorubicin (▪) and after a 7Gy irradiation (▪) or not (NT,□). IL-10 and TNFα levels were measured by ELISA on 100 µL supernatants as described in materials and methods. Results were expressed in pg/mL for 10^8^ cells at day of the assay per 24 h as the concentration in experiments performed in duplicate. The p value was determined according to a paired T-test. (C,D) Correlation between IL-10 and TNFα secretion and resistance to treatment. These box-plots compiled data from ELISA assays described in (A,B) and cell response to treatment showed in Figure 1. We divided cells into two groups, cells which were resistant to radiations (▪) as well as doxorubicin (Daudi, U266, and Namalwa cells whose proliferation >50% after 48 h treatment) and U937 sensitive cells whose proliferation <50% after 48 h treatment (□). The p value was calculated according a Wilcoxon statistical test.

Thus we measured the level of other cytokines potentially involved in treatment resistance like IL-10 and TNFα [34], [35]. IL-10 and TNFα are well-known growth factors for lymphoma and myeloma cell lines [36], [37]. First, we could notice that cells resistant to treatments (U266, Daudi and Namalwa cells) secreted a high level of these cytokines, particularly TNFα for U266 and Daudi cells and IL-10 for Namalwa cells, in marked contrast with the sensitive U937 cell line, whose production was limited (Figure 8A and 8B). Cytokine secretion strongly correlated with cell resistance to treatments, as revealed by the box-plots in Figure 8C and 8D (p<0.05). Interestingly, doxorubicin enhanced TNFα secretion by Daudi cells, contrary to radiations which increased IL-10 release by these cells. Similar results were obtained for Namalwa cells, but in a non significant manner. The observation of a correlation between baseline and anticancer treatment-induced production of TNFα and IL-10 with resistance suggests that multiple cytokines contribute to resistance in these cell lines, as described previously in our lab [17].

## Discussion

This study revealed that a potent correlation exists between IL-6 expression by oncohaematologic cells and their resistance to conventional treatments: U266, BL-36, Daudi and Namalwa cells expressing IL-6 constitute the most resistant cells to radiations and doxorubicin (Table 1 and Figure S1, S2). IL-6 inhibition successfully interfered with IL-6 induced cell signaling pathways in U266 and U937 cells, and particularly reduced STAT3 phosphorylation on Tyr705, on its own and in the presence of doxorubicin as cisplatin, or following irradiation at 7Gy (Figure 2 and Figure 4). In contrast, IL-6 inhibition marginally affected the level of p-STAT3 on Ser727. The function of this phosphorylated residue is always nowadays much debated: some labs suggested that this phosphorylation was required for STAT homo-dimerization and DNA binding [38], contrary to others supporting its role for the transcriptional activation of its targets through its association with Pin1 and p300 [39], [40]. Chung *et. al* showed that the phosphorylation of STAT3 on Ser727 is due to p42/p44 and that an inverse correlation between Ser727 phosphorylation and that on Tyr 705 exists [41]. Although p-p42/p44 level was impaired through IL-6 inhibition in U266 and Daudi cells (Figure 2A and Figure 3, respectively), the level of p-STAT3-Ser727 was increased in U266 cells after 30 and 60 min exposure to IL-6, but was also elevated in U266 and U937 cells following radiation or chemotherapy treatments (Figure 4). This suggests that 1) other kinases than p42/p44 regulate STAT3 phosphorylation on Ser727 in our models, and that 2) this hyperphosphorylated form of STAT3 could be more active for the transcription of its targets.

Daudi and Namalwa cells were unresponsive to endogenous as exogenous IL-6 signal, as revealed by western-blot analysis through p-STAT3 measurement (Figure 2C and data not shown). Nevertheless, a reduced level of phospho-p42/p44 was observed in Daudi cells after a brief exposure to IL-6 associated to anti-IL-6 signals if exogenous gp80 was concomitantly added (Figure 3). A similar observation has been recently made for U266 cells exposed to the proteasome inhibitor bortezomib® combined to the NFκB inhibitor 6-amino-4-quinazoline [42]. These two agents only efficiently interfered with IL-6 induced phosphorylation of STAT3 and p42/p44 if soluble gp80 was added.

Although resistant cells responded to radiations as well as cytotoxics by an IL-6 release (Figure 7), an anti-IL-6 antibody was poorly efficient to counteract cell resistance, as revealed by the measurement of cell growth and apoptosis (Figure 6, Figure S3, and Figure S5). Altogether, it seems that IL-6 inhibition combined to anticancer treatments efficiently negates IL-6 signaling for short time but looses its potency on general physiological processes after a longer. This could be linked to a counterbalanced mechanism induced by doxorubicin and ionizing radiations, resulting in expression and secretion of the agonist receptor gp80 (Figure 7C). Thus, a potent therapeutic alternative could be the association of an anti-IL-6 with an anti-IL-6R like tocilizumab, as suggested by the improved inhibitory effect of anti-IL-6 associated to anti-gp80 on Daudi cell growth (Figure 7D). A work of Mizutani *et al*. strengthened this idea, since an anti-IL-6R sensitized RCC to cisplatin [16].

We could not rule out that IL-6 acts as an intracrine growth factor in the cells we selected, since an old study showed that an anti-IL-6 antibody did not inhibit U266 and RPMI8226 cell growth [43]. Although anti-IL-6 combined to exogenous IL-6 could abrogate IL-6 signaling pathways in U266 and Daudi cells (Figure 2 and Figure 3), silencing IL-6 through the use of RNA technology could confirm this hypothesis. Such an intracrine loop has been previously identified in our lab for RCC, through the use of antisense oligonucleotide [3], and corroborated by the anti-IL-6 antibody inefficiency on radioresistant RCC. A previous study by Mizutani and coworkers on RCC cells similarly showed that anti-IL-6 as anti-IL-6R antibodies were only efficient on freshly patient derived RCC in association with cisplatin [16], through the down-regulation of Glutathione S Transferase-π (GST) mRNA. In our case, GST is not necessarily implicated in cell resistance to doxorubicin or radiations. Finally, we could not ignore the essential impact of tumour environment on cell survival and growth and could imagine that IL-6 inhibition through the use of anti-IL-6 antibodies could block cytokine pools secreted by the neighboring cells and thus could reveal a potent benefit *in vivo*.

Despite IL-6 involvement in cancer cell resistance was well established for various types of cancers [19], [21], this cytokine did not set itself up as the main actor of radio-and chemoresistance of Daudi and Namalwa cells, since they were weakly sensitive to anti-IL-6 antibodies. Two previous studies examined the effect of an anti-IL-6 in association with cisplatin on cancer cells. Dedoussis *et al.* showed that an anti-IL-6 combined to cisplatin exerts a synergistic cytotoxic effect (only around 20%) on K562 erythro-leukemic cells [19]. Borsellino *et al.* revealed that an anti-IL-6 antibody efficiently promotes a cytostatic effect on prostate cancer cells exposed to cisplatin in a sequential dependent manner (around 40% cytotoxicity) [44]. Thus, IL-6 seems to be partially involved in chemoresistance and only for a panel of agents, as suggested by the difference of cell behaviour in response to cisplatin and doxorubicin in our work: no release of IL-6 in response to cisplatin contrary to doxorubicin (Figure 7).

However, a lack of efficiency for antibody based treatment plus cisplatin had been previously observed for Rituximab on Daudi cells [14]. This study revealed that the anti-CD20 antibody has no effect on Daudi and Ramos cells but acts synergistically with cisplatin on the two non-Hodgkin's lymphoma 2F7 and 10C9 cells, resulting in the down-regulation of IL-10, another cytokine activating STAT family proteins and involved in cell resistance to treatment. IL-10, as IL-6, is a growth factor for non Hodgkin's lymphoma [37] and is often correlated to a poor prognosis [45]. Otero *et al.* suggested that B cells were resistant to ionizing radiations through a process involving IL-6 and IL-10, since knock-out mice for IL-10 as IL-6 become more sensitive to ionizing radiations. Finally, IL-10 inhibits the pro-apoptotic effect of doxorubicin on Daudi cells [17]. In this way, our study showed that high quantity of IL-10 was secreted by resistant cells (U266 and particularly Daudi and Namalwa cells) (Figure 8). We could envisage a counterbalanced effect of this cytokine in response to IL-6 inhibition. A close link between IL-10 and IL-6 had been previously observed for cell signaling and more particularly concerning STAT3 and SOCS3. IL-10 is able increasing SOCS3 expression in neutrophils [46] and enhancing STAT3 phosphorylation in macrophages for a longer period than IL-6 [47]. We could hypothesize IL-10 implication for maintaining STAT3 phosphorylation at a high level in Daudi and Namalwa resistant cells. Thus, silencing of IL-10 mRNA or of common signaling mediators such as STAT3 could allow highlighting the interconnection between these two cytokine pathways.

Altogether, these data suggest that oncohaematologic cell response to anticancer treatments like doxorubicin or ionizing radiations involves a complex network organized around three main cytokines, IL-6, IL-10 and TNFα, which could be differently activated according to the cell and the treatment. Interfering with one member of this network, like IL-6 in our study, seemed to induce counteracting signals in refractory cells, in attempt to maintain their proliferation and anti-apoptotic signaling pathways at a certain level of activation. In consequent, a more precise knowledge of cytokines and pro-inflammatory proteins engaged in response to treatment-induced death signals is necessary to identify the best anticancer therapeutic protocol.

## Materials and Methods

### Cell culture

Human cancer cells were obtained from ATCC (Manassas, VA, USA): Daudi: CCL-213, U266: TIB-196, RPMI 8226: CCL-155, U937: CRL-1593.2, Rs4; 11: CRL-1873, Raji: CCL-86, BL-36: CCL-87, Ramos: CRL-1596, BJAB: HB-136 and Namalwa: CRL-1432. Cells were grown in RPMI1640 (Gibco, Carlsbad, CA, USA), supplemented with 10% decomplemented fetal calf serum (Lonza, Basel, Switzerland), 10 mL penicillin streptomycin (10 U/mL/10 µg/mL, Gibco, Carlsbad, CA, USA) and 5 mL L-glutamin (200 mM, Gibco, Carlsbad, CA, USA) at 37°C humidified atmosphere containing 5% CO_2_.

### Cell treatments

Cells were exposed to different chemotherapeutic agents doxorubicin (Teva, Jerusalem, Israel), taxol (Bristol-Myers-Squibb, Seattle, WA, USA), vinblastine (EG Labo, France) at 0.01, 0.1 and 1 µg/mL, or vincristine (Foulding, Salisbury, South Australia) and cisplatin (Merck, Darmstadt, Germany) at 0.1, 1 and 10 µg/mL. Cells were also exposed to 10 µM dexamethasone (Dex, Merck, Nottingham, UK). Cells were also exposed to 10, 1 or 0.1 µg/mL of murine anti-IL-6 B-E8 antibody purchased from Diaclone (Besançon, France) as compared to the control mouse IgG1 (B-Z1) (Diaclone, Besançon, France). Cells were also exposed to 10 µg/mL of a mouse anti-gp80 IgG1 (B-R6, Diaclone, Besançon, France). In signaling experiments, cells were exposed to 10 ng/mL IL-6 (ebioscience, San Diego, CA, USA) and 50 ng/mL recombinant IL-6 receptor gp80 or IL-2 receptor (Peprotech, Rocky Hill, NJ, USA).

### RT–PCR

Total RNA was extracted from 5×10^6^ cells with Trizol® (Invitrogen, Carlsbad, CA, USA). One microgram of total RNA was subjected to reverse transcription by using oligodT and susperscript reverse transcriptase (Invitrogen, Carlsbad, CA, USA). Amplification of genes of interest IL-6, IL-6R and gp130 was performed with primers designed by Eurogentec (Seraing, Belgium) as follows: IL-6R-forward : CAT-TGC-CAT-TGT-TCT-GAG-GTT-C, reverse: AGT-AGT-CTG-TAT-TGC-TGA-TGT-C; gp130-forward: AGC-CAG-ATT-CCT-CCT-GAA-GAC-A, reverse: AAG-GTT-TAA-GGT-CTT-GGA-CAG-TGA-A; IL-6-forward: GTA-CAT-CCT-CGA-CGG-CAT-CTC, reverse: AGC-CAT-CTT-TGG-AAG-GTT-CAG. PCR was performed for 50 cycles with 1 min30 in denaturating conditions at 94°C, 2 min annealing at 55°C and finally 1 min 30 extension at 72°C. Results were analyzed by 1.5% agarose gel electrophoresis.

### RT–quantitative PCR

cDNA obtained as previously was submitted to RT-qPCR in SYBR (Eurogentec, Seraing, Belgium): 5 min at 95°C followed by 40 cycles with 15 s at 95°C and 1 min at 60°C. The primers used were purchased from Eurogentec: IL-6-forward: GTA-CAT-CCT-CGA-CGG-CAT-CTC, reverse: AGC-CAT-CTT-TGG-AAG-GTT-CAG; SOCS3-forward: TGG-GAC-GAT-AGC-AAC-CAC-AA, reverse: CGA-AGT-GTC-CCC-TGT-TTG-GA, as compared to the tubulin A reference, forward: CCC-CTT-CAA-GTT-CTA-GTC-ATG-C, reverse: CAT-TGC-CAA-TCT-GGA-CAC-C.

### ELISA

IL-6, soluble gp80 (sgp80) and soluble gp130 (sgp130) levels were measured in 100 µL supernatants by high sensitivity IL-6, sIL6R and sgp130 ELISA kits from Diaclone (Besançon, France), respectively. Results were recorded with a MRX ELISA microplate reader (Dynex Technologies, Chantilly, VA, USA). IL-10 and TNFα secretion were measured in 100 µL supernatants obtained as previously with a kit from BenderMedsystem (Vienna, Austria).

### IL-6, IL-6R, and gp130 detection by flow cytometry

For detection of membranous gp130 and gp80, cells were incubated in serum albumin bovine 10% 30 min at room temperature, then with 1 µg/mL B-R3 (gp130 antigen), B-R6 (gp80) or B-A11 (CD45) antibodies (Diaclone, Besançon, France), 30 min at 4°C. The revelation was achieved by using a secondary antibody Goat anti-Mouse conjugated to phycoerythrin (PE) (Imgene, San Diego, CA, USA) during 30 min at 4°C. The isotype controls B-Z1 and B-Z2 (Diaclone, Besançon, France) were used at the same concentration for gp80 and gp130, respectively. For intracytoplasmic detection of IL-6, cells were incubated during 5 h with Golgi Plug (BD biosciences, San Diego, CA, USA). Then, 2×10^5^ cells/well were incubated in paraformaldehyde 10% 20 min at 4°C, following with 10 µg/mL B-E8 antibody (Diaclone, Besançon, France) 30 min at 4°C, with B-Z1 isotype as a control and finally with a secondary antibody Goat anti-Mouse conjugated to PE during 30 min at 4°C.

### Proliferation assay

Cells were irradiated at different doses between 7 and 30Gy, or exposed to the different chemotherapeutic drugs. Cells were resuspended into fresh medium and plated in 96 well plates at 5000 cells/well. Cell growth was measured 24, 48 and 72 h later by two methods:

#### With an ATP-based assay

Twenty µL of Cell Titer Glo luminescent reagent (Promega, Madison, WI, USA) was added in 100 µL cell suspension for 10 min. Luminescence was recorded using a Microbeta reader (PerkinElmer, Fremont, CA, USA).

#### With thymidine incorporation assay

Tritiated thymidine (GEhealthcare, Chicago, IL, USA) was added during 16 h after 24, 48 and 72 h treatment. Counting was also recorded by the Microbeta reader.

### Apoptosis detection

#### Annexin V/propidium iodide

The level of living and apoptotic/necrotic cells was revealed using annexin V-FITC/propidium iodide (PI) kit (Immunotech, Villepinte, France). 2×10^5^ cells were incubated with 1 µL annexin V in 200 µL binding buffer 1X on ice and measurement at FL1-H was performed 10 min later. Five microliters of propidium iodide were then added to cells and measurement at FL2-H was realized.

#### Caspase activity

Two tests were used to measure caspase activity. Total caspase activity was determined through the use of CaspACE FITC-VAD-FMK in situ Marker (Promega). In brief, 10 µM reagent was added to 10^5^ cells 20 min at 37°C before detection by flow cytometry. Activity of caspases 3 and 7 was also determined by Caspase-Glo 3/7 assay (Promega). 100 µL of the luminogenic substrate of caspases Ac-DEVD-pNA was added to 100 µL cell suspension for 1 h. Luminescence was recorded by the Microbeta reader.

### Western blot analysis

Pelleted cells were resuspended in lysis buffer (Tris 50 mM pH 7.4, NaCl 250 mM, EDTA 5 mM, NaF 50 mM, Triton X-100 0.1%, orthovanadate 1 µM) plus protease inhibitors for 30 min on ice. After a centrifugation at 14000 rpm for 10 min, supernatants were boiled for 5 min in Laemmli sample buffer (Biorad, Hercules, CA, USA). Analysis of protein content was performed on 4–12% gradient gel. After electrophoretic separation, 50 µg proteins were electrotransferred on a polyvinylidene difluoride membrane (Immobilon P, Millipore corp., Bedford, MA, USA). The membrane was then blocked for 1 h at room temperature with blocking agent 0.2% in PBS/Tween 0.1%, probed overnight with a primary rabbit antibody against the protein of interest, and finally revealed with a secondary anti-rabbit antibody HRP conjugated (Upstate Biotechnology, Lake Placid, NY, USA) and ECL Advance system (GEhealthcare, Chicago, IL, USA). Primary antibody used was obtained from Cell Signaling (New England Biolabs, Beverly, MA, USA): phospho-p42/p44 (reference 9101), p42/p44 (4695), phospho-STAT3 (Tyr 705) (9145), phospho-STAT3 (Ser 727) (9134) and STAT-3 (4904), used at 1/1000 to 1/200.

### Statistical analysis

Statistical analysis of the differences between various treatment and control groups was done using a Student's T test or a Wilcoxon test. P<0.05 was considered statistically significant.

## Supporting Information

Figure S1 Expression of gp130, gp80 and IL-6 in U266, Daudi, Namalwa and U937 cells. (A) One microgram total RNA was subjected to RT–PCR for the detection of IL-6, gp130, and gp80, as described in materials and methods with β_2_µglobulin as a control. (B) Membrane staining of gp130 and gp80 or intracellular staining of IL-6 was performed on 2×10^5^ cells as described in materials and methods. The isotype control was shown as an empty curve. Numbers indicate the percentage of positive cells. Data shown were representative of two independent experiments. (C) IL-6, sgp130, and sgp80 levels were measured by ELISA on 100 µL supernatants as described in materials and methods. Results were expressed as the mean ± S.D of three independent experiments realized in duplicate (pg/mL).
(0.18 MB TIF)

Figure S2 ATP-based proliferation assay was consistent with thymidine incorporation assay. Daudi cell proliferation was measured as described in Materials and Methods by two assays, an ATP-based assay (empty line) or thymidine incorporation assay (dotted line), at time 24, 48, and 72 h. Results were represented as the mean of two independent assays realized in duplicate.
(0.05 MB TIF)

Figure S3 IL-6 inhibition in combination with 7Gy radiations or doxorubicin poorly affected Daudi cell proliferation measured by a tritiated thymidine test. (A) Cells were irradiated at 7Gy or not (NI). After irradiation, cells were resuspended into fresh medium, plated in 96 well plates at 5,000 cells/well and exposed to 10 µg/mL IgG1 (▪) or anti-IL-6 (▪) or vehicle (□). Cell growth was measured 72 h later with 20 µL reagent for 10 min. (B) Cells were treated for 48 h with 0.1 µg/mL doxorubicin in the presence of 10 µg/mL IgG1(▪) or anti-IL-6 (▪). Results were expressed as relative proliferation = number of treated cells at t time/number of cells at t0 in control conditions±S.D and represented a significant experiment among two realized in duplicate. The p value was determined according to a paired T-test * p<0.05, **<0.01. (C) Cells were exposed or not to 0.1 µg/mL IgG1 or anti–IL-6 antibody for 72 h, then irradiated at 7Gy (▪) or not (NI,□) and treated as previously for 72 h.
(0.06 MB TIF)

Figure S4 U937 and Daudi cells were sensitive to IL-6 inhibition following radiations and in the presence of dexamethasone, respectively. (A) IL-6 protected U937 cells from radiation-induced cytotoxic effects. U937 cells were exposed or not (NT) to IL-6 10 ng/mL in the presence of 10 µg/mL IgG1 or anti–IL-6 for 72 h, then irradiated (▪) or not (NI,□) and treated in the same conditions than previously for 72 h. Results were expressed as relative proliferation normalized to t0. (B) A combination of dexamethasone and anti-IL-6 effectively blocked Daudi cell proliferation. Daudi cells were treated or not with 0.1 µg/mL doxorubicin in the presence of 1 µg/mL anti–IL-6 and 10 µM dexamethasone for 48 h. Results were expressed as % of proliferation 48 h after treatment normalized to number of dexamethasone treated cells.
(0.07 MB TIF)

Figure S5 Anti–IL-6 did not affect radiation-induced caspase activity. Daudi cells were treated or not (NT) with 10 µg/mL IgG or anti–IL-6 for 72 h, irradiated at 7Gy or not (NI) and then treated as before radiations for 72 h. (A) Total caspase activity was measured by flow cytometry with a caspase inhibitor labelled with FITC. The isotype control was shown as a green line and untreated conditions in red line. (B) Caspase 3 and 7 activity was determined by a luminogenic caspase substrate as described in Materials and methods. Results were represented as fold caspase activity induction normalized to the t0 time and represented the most significant experiment among two realized in duplicate.
(0.09 MB TIF)
