# Supplementary figures and images for: Lymphoma and Myeloma Cell Resistance to Cytotoxic Agents and Ionizing Radiations Is Not Affected by Exposure to Anti–IL-6 Antibody
Source: PLoS One. 2009 Nov 30;4(11):e8026. doi: 10.1371/journal.pone.0008026 (PMC2779452; doi:10.1371/journal.pone.0008026)

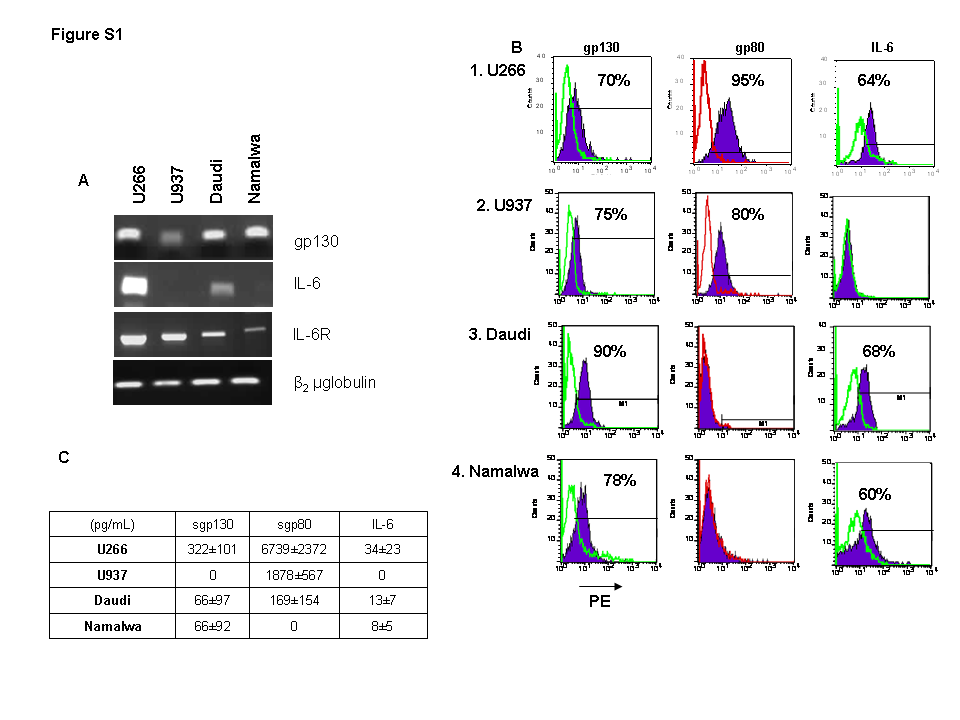

Supplement: Figure S1 — Expression of gp130, gp80 and IL-6 in U266, Daudi, Namalwa and U937 cells. (A) One microgram total RNA was subjected to RT–PCR for the detection of IL-6, gp130, and gp80, as described in materials and methods with β2µglobulin as a control. (B) Membrane staining of gp130 and gp80 or intracellular staining of IL-6 was performed on 2×10^5^ cells as described in materials and methods. The isotype control was shown as an empty curve. Numbers indicate the percentage of positive cells. Data shown were representative of two independent experiments. (C) IL-6, sgp130, and sgp80 levels were measured by ELISA on 100 µL supernatants as described in materials and methods. Results were expressed as the mean ± S.D of three independent experiments realized in duplicate (pg/mL). (0.18 MB TIF) [file pone.0008026.s001.tif]

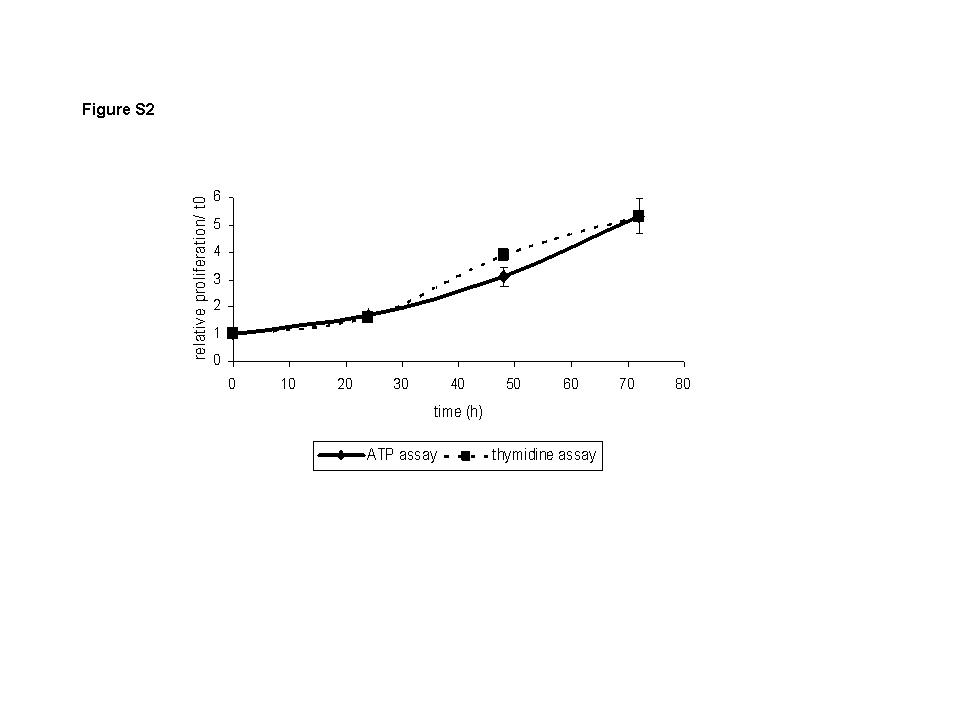

Supplement: Figure S2 — ATP-based proliferation assay was consistent with thymidine incorporation assay. Daudi cell proliferation was measured as described in Materials and Methods by two assays, an ATP-based assay (empty line) or thymidine incorporation assay (dotted line), at time 24, 48, and 72 h. Results were represented as the mean of two independent assays realized in duplicate. (0.05 MB TIF) [file pone.0008026.s002.tif]

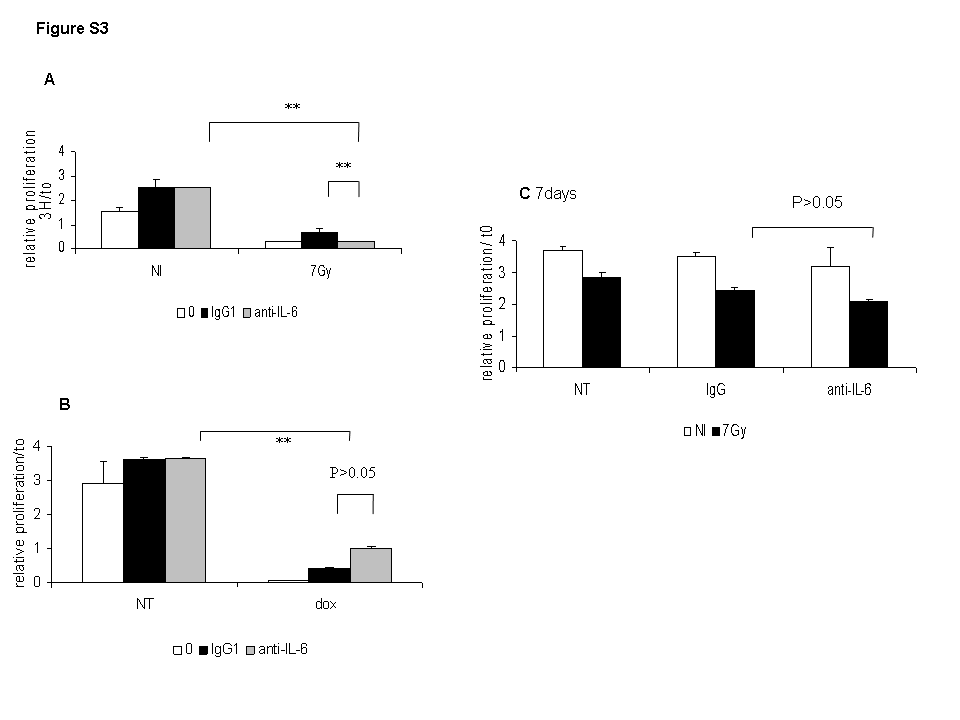

Supplement: Figure S3 — IL-6 inhibition in combination with 7Gy radiations or doxorubicin poorly affected Daudi cell proliferation measured by a tritiated thymidine test. (A) Cells were irradiated at 7Gy or not (NI). After irradiation, cells were resuspended into fresh medium, plated in 96 well plates at 5,000 cells/well and exposed to 10 µg/mL IgG1 (▪) or anti-IL-6 (▪) or vehicle (□). Cell growth was measured 72 h later with 20 µL reagent for 10 min. (B) Cells were treated for 48 h with 0.1 µg/mL doxorubicin in the presence of 10 µg/mL IgG1(▪) or anti-IL-6 (▪). Results were expressed as relative proliferation = number of treated cells at t time/number of cells at t0 in control conditions±S.D and represented a significant experiment among two realized in duplicate. The p value was determined according to a paired T-test * p<0.05, **<0.01. (C) Cells were exposed or not to 0.1 µg/mL IgG1 or anti–IL-6 antibody for 72 h, then irradiated at 7Gy (▪) or not (NI,□) and treated as previously for 72 h. (0.06 MB TIF) [file pone.0008026.s003.tif]

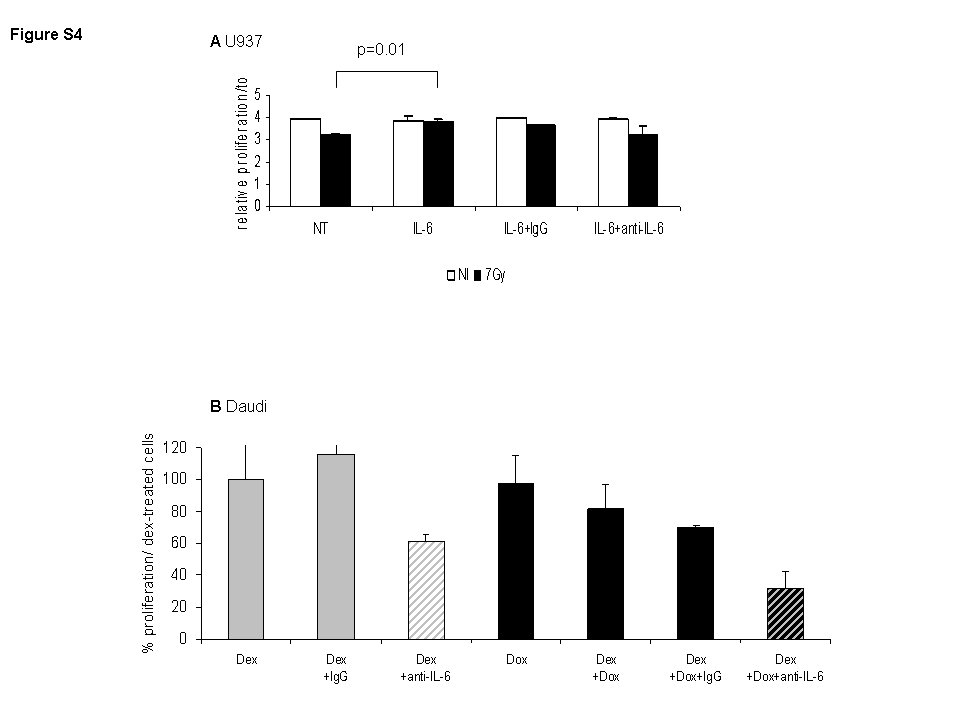

Supplement: Figure S4 — U937 and Daudi cells were sensitive to IL-6 inhibition following radiations and in the presence of dexamethasone, respectively. (A) IL-6 protected U937 cells from radiation-induced cytotoxic effects. U937 cells were exposed or not (NT) to IL-6 10 ng/mL in the presence of 10 µg/mL IgG1 or anti–IL-6 for 72 h, then irradiated (▪) or not (NI,□) and treated in the same conditions than previously for 72 h. Results were expressed as relative proliferation normalized to t0. (B) A combination of dexamethasone and anti-IL-6 effectively blocked Daudi cell proliferation. Daudi cells were treated or not with 0.1 µg/mL doxorubicin in the presence of 1 µg/mL anti–IL-6 and 10 µM dexamethasone for 48 h. Results were expressed as % of proliferation 48 h after treatment normalized to number of dexamethasone treated cells. (0.07 MB TIF) [file pone.0008026.s004.tif]

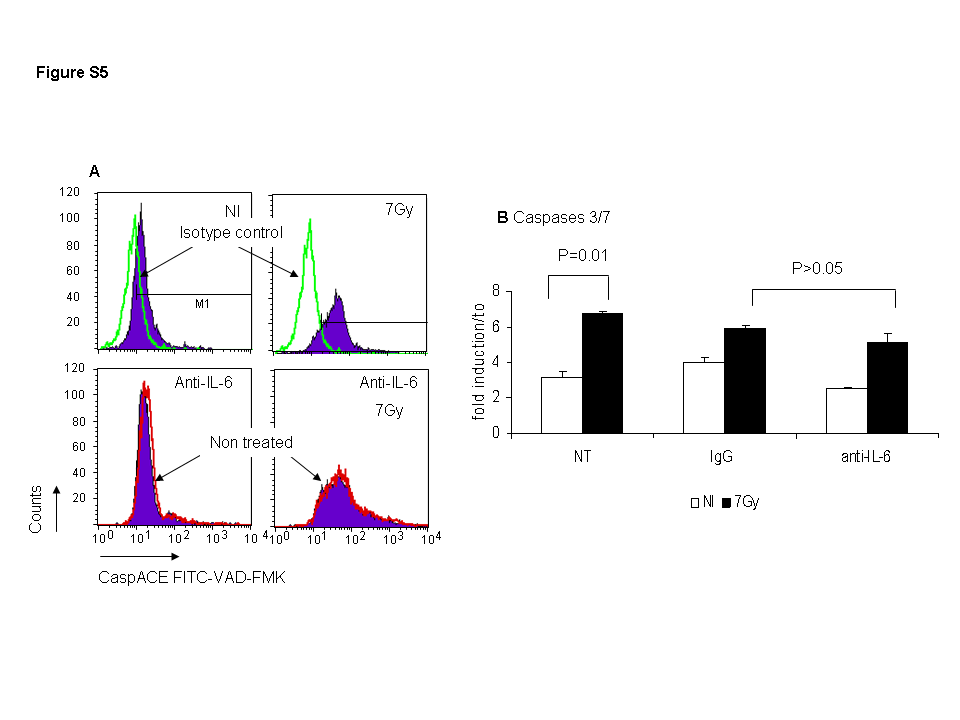

Supplement: Figure S5 — Anti–IL-6 did not affect radiation-induced caspase activity. Daudi cells were treated or not (NT) with 10 µg/mL IgG or anti–IL-6 for 72 h, irradiated at 7Gy or not (NI) and then treated as before radiations for 72 h. (A) Total caspase activity was measured by flow cytometry with a caspase inhibitor labelled with FITC. The isotype control was shown as a green line and untreated conditions in red line. (B) Caspase 3 and 7 activity was determined by a luminogenic caspase substrate as described in Materials and methods. Results were represented as fold caspase activity induction normalized to the t0 time and represented the most significant experiment among two realized in duplicate. (0.09 MB TIF) [file pone.0008026.s005.tif]
